# Biotransformation of Flavonoid Conjugates with Fatty Acids and Evaluations of Their Functionalities

**DOI:** 10.3389/fphar.2017.00759

**Published:** 2017-11-02

**Authors:** Cynthia Q. Sun, Keryn D. Johnson, Herbert Wong, L. Y. Foo

**Affiliations:** Integrated Bioactive Technologies, Research and Technical Services, Callaghan Innovation, Lower Hutt, New Zealand

**Keywords:** flavonoid conjugates, biotransformation, omega-3 fatty acids, grapefruit extracts, VEGF

## Abstract

Enzymatic conjugation with fatty acids including omega-3 polyunsaturated fatty acids (ω-3 PUFAs) derived from fish oil to three citrus fruit-derived flavonoids: grapefruit extract, naringin, and neohesperidin dihydrochalcone were investigated. The conversions were achieved over 85% under the catalysis of lipase Novozyme 435 in acetone at 45°C at semi-preparative scale. The conjugates were purified via solvent partition and silica gel chromatography and achieved 90–98% in purity. The NMR analysis of the conjugates confirmed that the fatty acid carbon chain was linked onto the primary –OH group on the glucose moiety of the flavonoids. The purified flavonoid conjugates alongside their original flavonoids were analyzed for antioxidant activities via 2,2-diphenyl-1-picrylhydrazyl scavenging assay, and anti-peroxidation test via peroxide values measured during a 1-week fish oil storage trial. Vascular endothelial growth factor (VEGF) assay was conducted with 1, 10, and 100 μM of naringin and grapefruits and their conjugates, respectively, and total VEGF levels were measured at 24 and 48 h, respectively, using ELISA and dot blot analysis. The results from these functionality experiments demonstrated that flavonoid FA conjugates have at least comparable (if not higher) antioxidant activity, anti-peroxidation activity, and anti-angiogenic activity.

## Introduction

Bioactive natural compounds are produced in plants and animals for various metabolic purposes and have been consumed as functional foods or used in indigenous medicine throughout human civilization. Among these bioactives, flavonoids and polyunsaturated fatty acids (PUFAs) are two groups of compounds that have attracted worldwide scientific interest in the past 30 years. The results produced from this research suggested that many flavonoids possess not only antioxidant activity but also multiple immune-modulating properties (Xu et al., 2000; Castro-Vazquez et al., 2016). Evidence-based health claims have been made successfully for PUFA as cardiovascular protecting agents and anti-inflammatory agents (Zibadi et al., 2007; Dasilva et al., 2015; Shang et al., 2017). Natural sources that are rich in flavonoids and PUFA have been (or have the potential to be) utilized as bioactive ingredients in nutraceutical and pharmaceutical products. However, these extracts, might be restricted to the variety of applications, due to unsuitable physical properties in the downstream product development phase, i.e., PUFA is sensitive to heat and has limited shelf life due to prone to oxidation; flavonoids have moderate polarity and propensity to interact with biological materials (Hidari et al., 2009; Dasilva et al., 2017). Post extraction modification, aiming to overcome the limitations of the natural extracts, is an option for maximizing their applications.

Enzymatic esterification of antioxidants with fatty acids was firstly reported on ascorbic acid and palmitic acid (Humeau et al., 1995). This biotransformation created a novel ascorbic ester with much improved lipophilic solubility with equivalent antioxidant activity as ascorbic acid. Since then, a number of polyphenols derivatives have been made enzymatically (Guyot et al., 1997; Buisman et al., 1998; Stamatis et al., 2001), all of which proved to have different physical properties while maintaining the original bioactivities (Riva et al., 1998; de Pinedo et al., 2005, 2007; Burham et al., 2009).

Naringin and neohesperidin are two main flavonoids present in grapefruit extract (Murugan et al., 2009; Chanet et al., 2012). Neohesperidin dihydrochalcone (NHDC) is derived from neohesperidin via hydrogenation (Horowitz and Gentili, 1963) for its commercial applications as a food additive (sweetener) in dairy and other food preparations. As its parent compound, NHDC possesses antioxidant properties and is inhibitory to hypochlorous acid-induced DNA damage and protein degradation (Choi et al., 2007).

This study aimed to attach selected individual fatty acids (lauric acid, oleic acid, and linolenic acid) and a omega-3 (ω-3) PUFA rich mixture of fatty acids derived from fish oil to three citrus fruit-derived flavonoids: grapefruit extract, naringin, and NHDC, under lipase catalysis. Food grade fish oil was used as a source of ω-3 instead of pure individual ω-3 fatty acid. The novel flavonoid/fatty acid conjugates generated (a total of 10) were analyzed by LC–MS, purified on a chromatographic column, and structure-identified before investigated for their functionalities in lipid peroxidation protection, 2,2-diphenyl-1-picrylhydrazyl (DPPH) scavenging activity, and potential effects on angiogenesis via evaluation on vascular endothelial growth factor (VEGF) levels.

## Materials and Methods

### General

All chemical standards including free fatty acids and flavonoids are from Sigma (St. Louis, MO, United States). All solvents used for the solvent partition and silica gel chromatography are of analytical grade and from Scheduler (Barcelona, Spain). The HPLC grade solvents for LC–MS are from Merck (Darmstadt, Germany).

Grapefruit extracts were obtained in our previous research and contain approximately 40% naringin and 40% neohesperidin based on LC–MS analysis (unpublished work).

The mixture of ω-3 PUFA rich fatty acids was derived from fish oil (salmon) and was purified per method by Christie and Han (2010). Its FA composition was outlined in **Table 1**. The flavonoid esters with this mixture of FA was profiled via gas chromatography coupled with a mass spectrometer (GCMS-QP2010Ultra, Shimadzu) using a wax (polyethylene glycol) column.

**Table 1:** The FA profiles in the ω-3 PUFA rich mixture and NHDC PUFA esters.

Main fatty acids	Lipid name	FA mol % in the PUFA mixture	FA (MW)^∗^	NHDC ester (MW)^∗^	FA mol% in NHDC esters
Palmitic acid	C16:0	5.3	256.4	851	6.0
Palmitoleic acid	C16:1 (*n*-7)	2.3	254.4	849	–
Oleic acid	C18:1 (*n*-9)	10	282.5	877.1	13.2
Docosahexaenoic acid (DHA)	C20:5 (*n*-3)	27	302.5	897.1	35.2
Docosapentaenoic acid (DPA)	C22:5 (*n*-3)	4	330.5	925.1	6.2
Eicosapentaenoic acid (EPA)	C22:6 (*n*-3)	17.6	328.5	923.1	26
*Stearidonic acid (SDA*)	C18:4 (*n*-3)	3	276.5	871.1	4.4
Eicosatetraenoic acid (ETA)	C20:4 (*n*-3)	3	304.5	899.1	–
Stearic acid	C18:0	5	284.5	879.1	6.0
Eicosenoic acid	C20:1 (*n*-9)	2	310.5	905.1	3.1

*^∗^MW, molecular weight; –, lower than detection limit.*

### Enzymatic Conjugation of Flavonoids Esters

Grapefruit extracts, naringin, and NHDC (at 4 mg/ml in acetone, respectively) were esterified with free fatty acids (lauric acid, oleic acid, linolenic acid, and a mixture of FAs derived from PUFA rich fish oil, respectively), under the catalysis by Novozyme 435 (Novo Nordisk, Denmark). These enzymatic reactions were run in duplicate and in a temperature controlled orbital shaker (at 120 × *rpm* and at 45°C) for 3–5 days. Acetone was pre-dried with molecular sieves (4 Å, Sigma). Extra MS 4 Å were also added into the reaction mixture to control the water level.

According to the product specification from the manufacture, Novozyme 435 is an immobilized lipase from *Candida antarctica* B, with optimal working temperatures between 30 and 60°C^1^. The reactions conducted in this study were set to be at 45°C in compensation of PUFA’s sensitivity to heat. The enzyme has a claimed 10,000 PLU/g activity (1 PLU is the amount of enzyme activity which generates 1 μmol of propyl laurate per minute under defined standard conditions).

An aliquot (500 μl) of sample was withdrawn once daily and subjected to the HPLC analysis to monitor the esterification of flavonoids. The HPLC analysis was conducted on a quaternary pump system (Agilent 1100) with a gradient mixer, an auto-sampler, and a diode array detector (Agilent 1100). The separation of flavonoids and its esters were achieved using a C18 column (Luna C18, 5 μ, Phenomenex, 250 × 4.6 mm), under a gradient elution of 0.1% formic acid (A) and acetonitrile in 0.1% formic acid (B) at 35°C (0–10 min, 90–80% A; 10–15 min, 80–10% A; 15–24 min, 10% A; 24–26 min, 10–90% A; 26–30 min, 90% A). The chromatography was monitored at the UV wavelength of 280 nm. The conversion was calculated as the reverse percentage of flavonoids left in the reaction at each sampling time-point.

After the biotransformation, the reaction mixture was filtered to remove the lipase and molecular sieve, then dried under rotary vacuum evaporation. The oily substance was phase-separated with heptane and acetonitrile at 60°C to remove unreacted FAs. The unreacted flavonoids and flavonoids FA esters in acetonitrile were dried and re-dissolved in acetone, then separated by silica gel chromatography (60 Å, 230–400 mesh, GE Life Science) under chloroform/methanol elution. The silica gel flash column was eluted with petroleum ether (60–80°C boiling point) first, then chloroform/methanol at 95:5 and at 90:10. The flavonoids FA esters were eluted with chloroform/methanol at 85:15, followed by vacuum dry. Finally, the column was cleaned with methanol to elute the un-reacted flavonoids.

Each fraction eluted from the silica flash chromatography was analyzed by a LC–MS (Waters 2795 LC Combined with a Q-Tof Premier Mass Spectrometer, Micromass, United Kingdom).

The flavonoids and its esters were eluted on a C18 column (Kinetex 2.6u, 100 Å, 50 × 3.0 mm) by a gradient of A: 0.1% formic acid in water, and B: acetonitrile in A (0–10 min, 20–40% B; 10–15 min, 40–90% B; 15–35 min, 90% B). Flow rate is at 0.3 mL/min and column temperature was maintained at 40°C.

Purified flavonoids esters (naringin lauryl and oleic esters, and NHDC mixture esters) were structure analyzed by both ^13^C and ^1^H NMR on a Bruker Avance 300 Spectrometer. Deuterium dimethyl sulfoxide (DMSO-D6) (Cambridge Isotope Lab, United Kingdom) was used as the solvent.

A summary of flavonoids FA esters synthesized in this study and the analysis and assays performed on them were listed in Supplementary Table S1.

### Antioxidant Property

The purified flavonoid conjugates alongside their original flavonoids were analyzed for antioxidant activities via DPPH (diphenyl-picryl-hydrazyl radical) scavenging assay. The synthetic free radical DPPH is a purple colored compound with absorption at 517 nm. The scavenging of this radical by a potential antioxidant would quench the color and can therefore be measured by the changes in absorbance at 517 nm (Blois, 1958).

All samples were dissolved in ethanol or in DMSO as stock solution then diluted with ethanol in the test. Ascorbic acid was used as the antioxidant control. An aliquot (100 μl) of each sample was added to 2 ml of DPPH solution (0.1 mM in ethanol). The mixture was left to react for 90 min in the dark then the absorbance of the mixture at 517 nm was measured. The antioxidant activity was assessed as the percentage of free radical scavenged (FRC) as follow:

% FRC = [*A* (blank) – *A* (sample)] divide by *A* (blank) × 100, where *A* is the absorbance at 517 nm. All samples were measured in triplicate.

### Lipid Peroxidation Protection Measurement

The Hoki fish oil was supplied from Bakels (Rothenburg, Switzerland). α-Tocopherol (Vit E) and butylated hydroxytoluene (BHT) were purchased from Sigma.

The flavonoids and their fatty acids esters (10 mg each) were added into 50 ml (∼45 g) fish oil, respectively, in a bottle wrapped with foil paper. The bottles were shaken at room temperature for 1 h, then left at 9°C in the dark. The experiment with fish oil was only used as negative control, and the experiment with fish oil and α-tocopherol or BHT was the positive control. The sampling was drawn daily (five times per week) and was left for 30 min at room temperature (20–25°C) before testing. Oxidative stability was determined by measuring the peroxide value and anisidine value. The experiments were run in duplicate.

The peroxide value is a measurement for primary oxidation products, e.g., hydro-peroxides. It is assessed via titration of liberated iodine with standardized sodium thiosulphate solution, according to the AOCS official method (AOAC 965-33 Titration, 1998a).

Formation of secondary oxidation products was assessed by the p-anisidine value, which was measured via the absorbance at 350 nm from the reactants between p-anisidine with aldehyde compounds present in oil, per standard method (AOCS official method Cd 18-90, 1998a).

### Sheep Aortic Ring – VEGF ELISA and Dot Blot Analysis

The method developed was based on the description in Baker et al. (2012). Sheep aorta were obtained from the local slaughter house (Taylor Preston, Wellington, New Zealand) and were processed within 2 h of collection. The fat was removed from the external layer of the aorta and the outside layer of the tissue sprayed with 70% ethanol. The aorta was cut into 1 cm rings using a sterile razor blade and placed into a 24-well cell culture plate. Then, 1 ml of sterile PBS was added, followed by the addition of test compounds at a final concentration of 1, 10, and 100 μM of the flavonoids and their conjugates, respectively. The total VEGF levels were measured at 24 h using dot blot analysis. The plates were incubated at 37°C before the solution was recovered and analyzed. Relative VEGF levels were compared to a PBS control. Samples were analyzed in replicates of six.

The dot blot analysis for VEGF content was conducted as following: A mouse VEGF standard curve was prepared in triplicate using 10-fold dilutions from 0.01 to 100 μg. Briefly, 0.5 μl of sample and standard was transferred onto a PVDF membrane. The membrane was blocked with 1% BSA solution in PBS buffer for 1 h at RT. The membrane was then probed with primary antibody mouse anti VEGF (Invitrogen VG1) at 1:500 in 0.1% BSA in PBS for 2 h. The membrane was washed three times with 5 ml of PBST (PBS containing 0.1% Tween 20). The secondary antibody goat anti-mouse alkaline phosphatase was prepared at 1:1000 dilution in PBS containing 0.1% BSA. The secondary antibody was allowed to bind for 1 h. The membrane was washed three times for 5 min with PBST and then finally with MilliQ water for 2 min before 5 ml of BCIP/NBT was added. The color was allowed to develop for 30–60 min. An image was taken and the density of the dots determined using NIH Image J and the average spot intensity calculated. A set area was defined which was of sufficient size to accommodate the spot on the dot blot (spot area 90). Black was defined as 255 and white as 0. The gray scale analysis of each spot was performed. The spot densities of the standard curve were used to calculate VEGF concentration. A Student’s *t*-test was performed to determine statistical significance.

## Results

### Enzymatic Conjugation of Flavonoid Fatty Acid Esters

**FIGURE 1:**
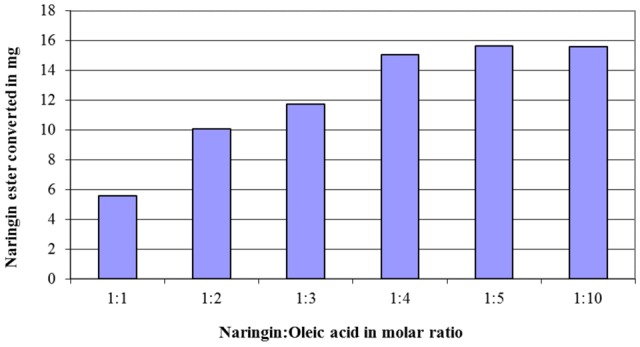
The effect of the molar ratio between naringin and oleic acid on the quantity of the naringin ester produced in a reaction with 20 mg naringin in 5 ml acetone at 45°C after 3 days (80 mg of Novozyme 435 was added at the start of the reaction).

**FIGURE 2:**
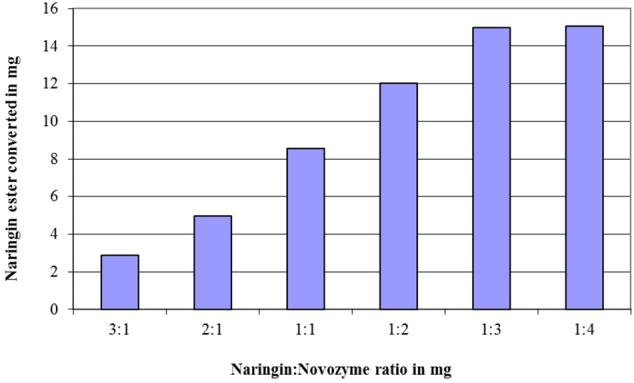
The effect of the mass ratio between naringin and Novozyme 435 added on the quantity of the naringin ester produced in a reaction with 20 mg naringin in 5 ml acetone at 45°C after 3 days. Oleic acid was used as the acyl donor substrate at a molar ratio of 5:1 versus naringin in the reaction.

The effects of the enzyme quantity and the amount of acyl donor (fatty acids) on the conversion of flavonoid esters were exemplified via the naringin (20 mg in 5 ml acetone) esterification with oleic acid. The results in **Figure 1** demonstrated a linear growth in conversion when molar ratio between fatty acids to naringin increased from 1:1 to 4:1, however, no further improvement after 5:1 ratio was observed. When this ratio was applied, the optimum amount of Novozyme 435 in the naringin esterification was shown to be at 3:1 (**Figure 2**).

**FIGURE 3:**
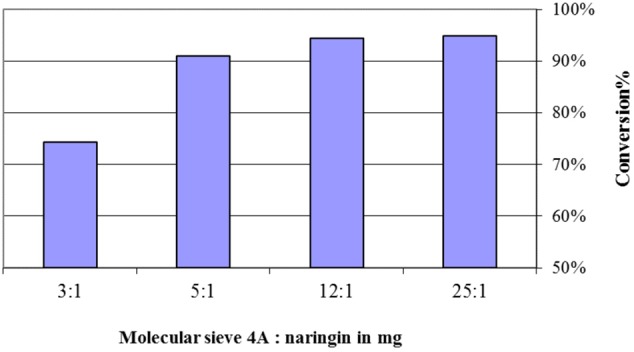
The effect of molecular sieve 4 Å added in the naringin esterification with oleic acid. The reactions contained 20 mg of naringin, 4 g of oleic acid, and 100 mg of Novozyme 435 in acetone (5 ml) at 45°C for 5 days.

Water is the byproduct of the flavonoid esterification. Molecular sieve 4 Å was used to control the level of water in this study. **Figure 3** presented a positive relationship between the mass ratio of molecular sieve versus naringin and its conversion, however, when this ratio was higher than 12:1, no further improvement in conversion was observed.

**FIGURE 4:**
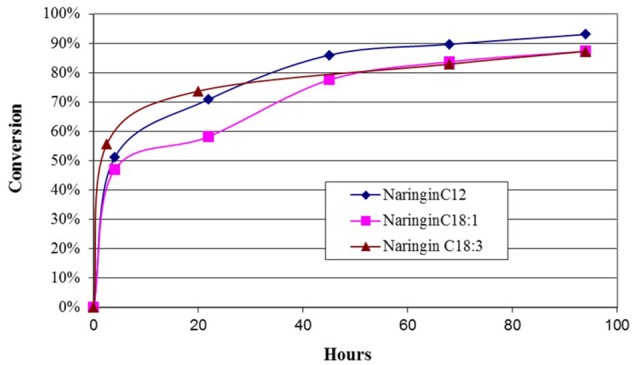
The time courses of the esterification of naringin with three fatty acids, respectively. The reactions contained 20 mg naringin and 35 mM either lauric acid, or oleic acid and linolenic acid in acetone (5 ml), respectively. Novozyme 435 was added at the beginning of the 4-day reaction at 45°C.

The reaction progress of the flavonoids esterification with different acyl donors is exemplified via naringin esterification with lauric acid, oleic acid, and linolenic acid, respectively. The results in **Figure 4** suggested that the esterification had a rapid start with a steep increase to 50% in conversion after 4 h, then slowed down but maintained a slow increase to near or slightly over 90% conversion over the next 90 h. Among different acyl donors, there are small differences in conversion rate over the 4-day period. Shorter carbon chain FA, lauric acid, had slightly higher conversion at the end of 4-day reaction (93%), while oleic acid and linolenic acid had similar conversions (88%).

In summary, the characteristic study on Novozyme 435 catalyzed esterification of flavonoids suggested that in a reaction containing naringin (4 mg/ml) at start, a ratio of 1:3 (mg/mg) between naringin and Novozyme, a ratio of 1:5 between naringin and oleic acid (in mol), and a ratio of 1:12 between naringin and molecular sieve 4Å (or 50 mg/ml acetone) are recommended to achieve the highest conversion from naringin to naringin oleic ester. The reactions were run at 45°C and over 3–5 days. These conditions were applied in the remaining reactions conducted in this study.

**FIGURE 5:**
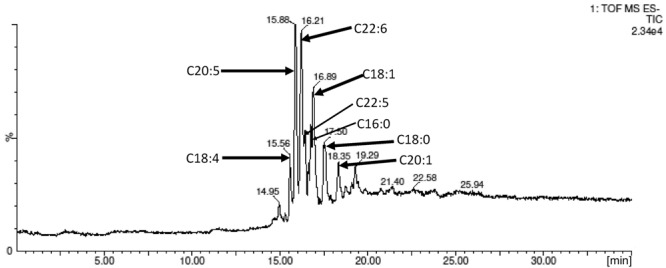
LC–MS analysis of the purified NHDC ω-3 PUFA esters. The cluster of ester peaks was identified with their respective M-1 (m/z) ions.

The semi-preparative scale esterification of naringin, NHDC, and grapefruit extracts (∼480 mg each in 120 ml acetone) were conducted with ω-3 PUFA derived from fish oil, respectively, over 4 days. HPLC analysis revealed a conversion of 91.9, 90.7, and 96.7%, respectively, for naringin, NHDC, and grapefruit extract (**Figure 5**). The acetonitrile–heptane extraction followed by silica gel chromatographic separation produced fractions of PUFA rich esters conjugated with naringin, NHDC, and grapefruit extracts, respectively.

A total of 10 flavonoids FA esters were made and purified in this study. For easy reference, all 10 esters were listed in Supplementary Table S1, with information of the analysis and assays performed on them.

The purity of these esters was analyzed by LC–MS. The results confirmed that purity of the new conjugates was at >90–98%. The main impurity was identified as the dissolved silica gel from the flash chromatography. Two small unknowns (<3% in total mass) peaks were identified by LC–MS as: methylated esters due to the usage of methanol dilution in the sample preparation for LC analysis; and oxidized (one -CH_2_ to -CO) esters in the case of linolenic acid esters.

**FIGURE 6:**
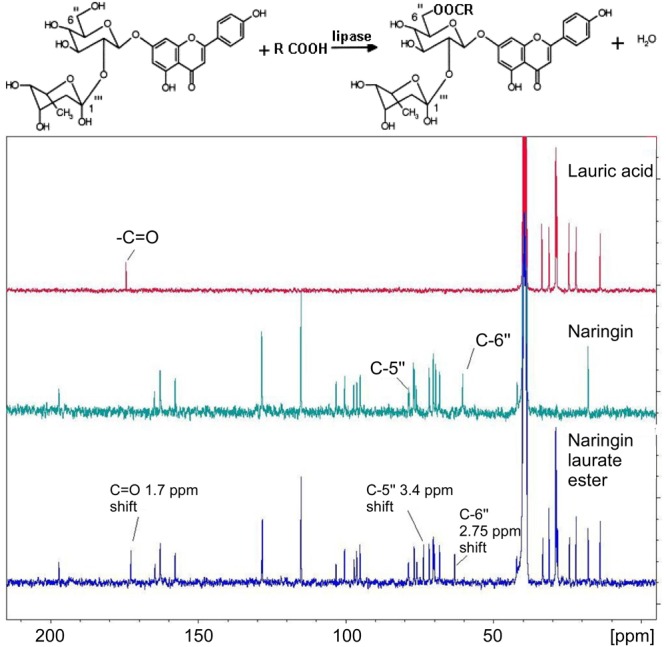
^13^C NMR spectrums of lauric acid (top-NMR), naringin (middle-NMR), and naringin laurate ester (bottom-NMR).

Naringin lauryl and oleic esters have been analyzed by both ^13^C and ^1^H NMR (300 MHz) in DMSO media. Two major shifts in the NMR data were observed on the naringin sugar moiety, specifically on the C″-6 of the glucose moiety (2.75 ppm shift) and C″-5 carbon (3.4 ppm shift). The data suggested a formation of ester bond, linking to the primary -OH on the glucose (**Figure 6**).

**FIGURE 7:**
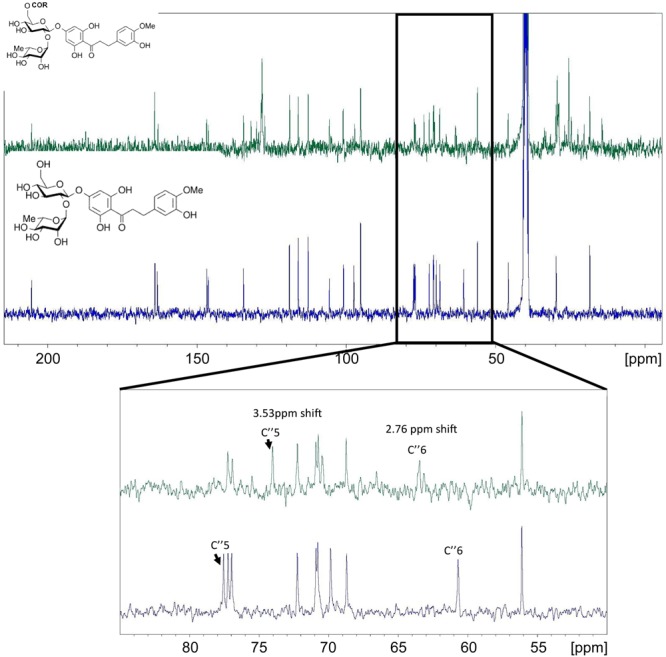
^13^C NMR spectrums of NHDC (bottom NMR) and its ω-3 rich PUFA esters (top NMR). The enlarged NMR graph is between 55 and 85 ppm.

Similar changes were observed from the NMR analysis of NHDC and its ω-3 PUFA esters (**Figure 7**). The C″-6 on the sugar moiety of NHDC had a 2.76 ppm shift and the C″-5 had a 3.5 ppm shift (**Figure 7**).

The FA profile in the ω-3 PUFA used in this study, obtained via GC-MS analysis, is shown in **Table 1**. It was found that DHA (C20:5) and EPA (C22:6) are the dominate PUFA, accounted for 27.0 and 17.6% of the total FA. The analysis of the main fatty acids in the flavonoids esters confirmed six main esters as DHA (C20:5), EPA (C22:6), DPA (C22:5), C18:1, C18:4, and C16:0; their molar distribution was consistent with the fatty acids composition found in the starting ω-3 PUFA rich FA mixture. This suggests no likelihood of selectivity for Novozyme 435 toward degree of saturation of FAs used in the enzymatic esterification.

### Antioxidant Property

**FIGURE 8:**
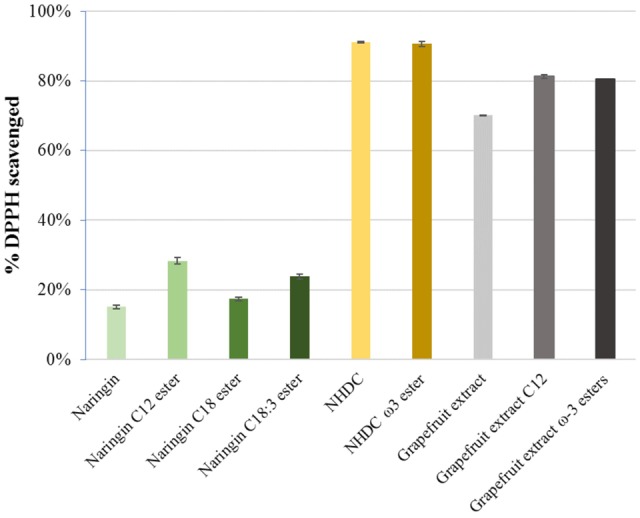
DPPH scavenging activities in naringin, NHDC, and grapefruit extracts and their FA esters; C12-lauric acid, C18:1-oleic acid, C18:3-linolenic acid, and ω-3 PUFA rich esters (each in 0.1 μmol).

The results from the free radical scavenging activity (DPPH) assay demonstrated that all flavonoids esters maintained at least same level of activity in comparison to their parent flavonoids, which are in line with the findings in studies for other polyphenolic esters (Buisman et al., 1998). The results suggest the esterification does not hamper the antioxidant activity of the parent molecules (**Figure 8**).

### Lipid Peroxidation Protection Measurement

**FIGURE 9:**
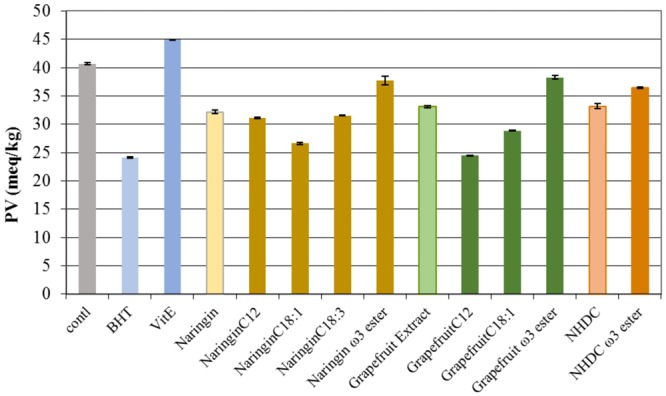
The peroxide values (PV) in oils incubated with naringin, NHDC, and grapefruit extract and their esters (in purified form, and 10 mg each in each experiment). BHT and Vit E were used as positive control. The negative control (control) had no added antioxidants. The values shown are at the end of 7-day trial (162 h). PV in oil is expressed as milli-equivalents per kg of oil (meq/kg).

The peroxide values of the fish oil incubated with each flavonoid and its esters, respectively, are graphed in **Figure 9**. All the compounds tested demonstrated protection against peroxidation in comparison to the control after 7-day storage. Naringin esters with C12, C18:1, and C18:3 showed better protection against peroxidation than naringin. In particular, naringin C18:1 ester and grapefruit C12 ester demonstrated anti-peroxidation strength equivalent to that found in BHT. However, all ω-3 PUFA esters had less activity than their flavonoid parent compounds – naringin, NHDC, and grapefruit extract, respectively. One could speculate that the tendency of PUFA itself to undergo oxidation might compromise the activity against peroxidation in flavonoid PUFA esters.

**FIGURE 10:**
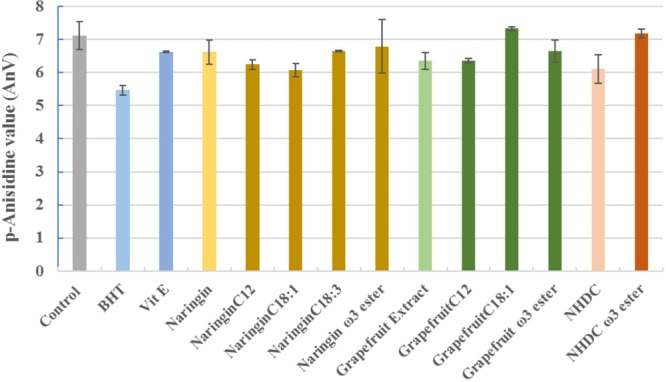
The *p*-anisidine values in oils incubated with naringin, NHDC, and grapefruit extract and their esters (in purified form, and 10 mg each in each experiment). BHT and Vit E were used as positive control. The negative control (control) had no added antioxidants. The values shown are at the end of 7-day trial (162 h).

The protection of flavonoids and their esters against the formation of secondary oxidative products are summarized in **Figure 10**. Except PUFA esters of naringin and NHDC, respectively, and grapefruit C18:1 esters, other tested compounds demonstrated Vit E equivalent activity against the formation of secondary oxidants.

### VEGF ELISA

**FIGURE 11:**
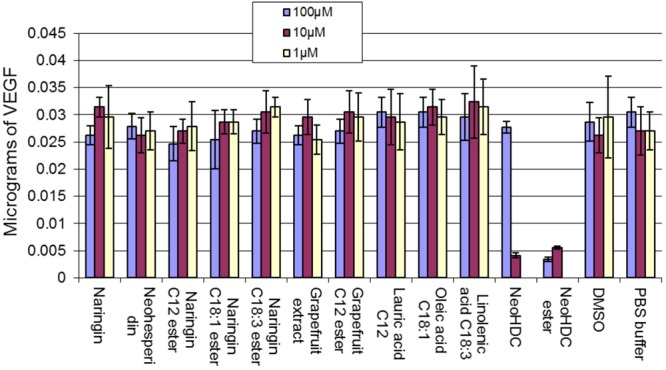
Effects of fatty acid ester and flavonoid conjugates on the production of VEGF from sheep aortic ring.

The results of VEGF expression in sheep aorta ring incubated with flavonoids and their esters are summarized in **Figure 11**. At the highest concentration tested (100 μM) naringin was shown to reduce VEGF levels compared to PBS control, which was significant (*p* < 0.005). The fatty acids alone (C12, C18:1, and C18:3) didn’t have any effect. Grapefruit extract 2 containing mainly naringin and neohesperidin also reduced VEGF levels (*p* < 0.01). However, when naringin was conjugated to various fatty acid esters there was a reduction in VEGF levels in a dose-dependent manner but this was not statistically significant, while the grapefruit lauryl ester had similar VEGF expression at 100 μM. This suggests that the anti-angiogenic potentials of naringin and grapefruit extract are at least partly retained after conjugation to fatty acids. The NHDC flavonoid when tested at 100 μM had a similar VEGF level as the PBS control whereas it was shown to be inhibitory when tested at 10 μM. The PUFA mixture ester of NHDC, however, was inhibitory at both concentrations tested.

## Discussion

Enzymatic esterification of hydrophilic polyphenols and flavonoids has advantages over chemical methods in regio- and enantioselectivity, with no side-reactions. As demonstrated in this research, the esterification of both naringin and NHDC with acyl fatty acids in different carbon chain saturation achieved high yields (>88%) under the catalysis of Novozyme 435. This approach was directly applied to the esterification of a crude natural product – grapefruit extracts, with a mixture ω-3 rich PUFA, on a semi-preparative scale. The results proved its feasibility with high conversion (>96%) in likely a food-grade application. The functionality analysis on this novel class of flavonoid esters in respect to antioxidant activity, lipid peroxidation protection, and VEGF expression in sheep aortic ring, confirmed that the novel conjugates had retained these functionalities from their parent flavonoid compounds. Longer experiment period on measurements for peroxide value and *p*-anisidine value, e.g., 4 weeks and 6–12 months, are recommended for more thorough investigation of the peroxidation protection activity of these novel conjugates.

In this study, a mixture of ω-3 rich PUFA, instead of individual ω-3 fatty acid, was chosen to make ω-3 flavonoid conjugates with an aim to develop a nutraceutical supplement. Although EPA and DHA the dominant components in the fish oil pure compounds were not used due to the economic considerations for this development. ω-3 PUFA has many health benefits to a wide range of diseases: asthma, depression, cardiovascular disease, ADHD, and autoimmune diseases, such as rheumatoid arthritis (Dasilva et al., 2015). However, its tendency to oxidation limits its shelf-life and applications. Flavonoids are good antioxidants and have been extracted from plants and fruits as nutraceutical supplements. A conjugate built with flavonoids and ω-3 PUFA will have an unique advantage over the two simply combined. With the presence of flavonoids, ω-3 PUFAs will have built-in protection against peroxidation; the presence of PUFA carbon chain will improve the lipophilic property in flavonoids, hence increase the penetration through the cell membrane. The flavonoid-ω-3 PUFA conjugates would have improved bioavailability than themselves alone, however, this remain to be tested.

Previously it has been shown by Lai et al. (2013) that citrus peel flavonoids have a potent anti-cancer effect through reduced expression of VEGF. Luo et al. (2008) also demonstrated that naringin had an ability to reduce the expression of VEGF in ovarian cancer cells. Schindler and Mentlein (2006) have shown that naringin reduced VEGF release from MDA human breast cancer cells. Our results report similar findings where naringin slightly reduced VEGF levels produced from aorta explants. Contrasting with these findings, however, others have shown that naringin stimulates wound healing and angiogenesis through up-regulation of mRNA expression of growth factor (IFG-1, TGF-β, and VEGF-c) (Kandhare et al., 2014). VEGF-c overexpression has been implicated in tumor angiogenesis (Tvorogov et al., 2010). In rats with spinal cord injury treatment with naringin at 20 or 40 mg/kg increased levels of VEGF expression suggesting that naringin is a neurotrophic factor under these conditions (Rong et al., 2012). A wound healing ointment containing 2% and 4% naringin has also been shown to stimulate collagen I synthesis and VEGF levels thereby promoting angiogenesis (Kandhare et al., 2016). Song et al. (2017) have also shown naringin’s ability to promote angiogenesis and bone healing through stimulation of VEGF/VEGFR-2 signaling pathway. The reason for these opposing findings is unknown but there could be a biphasic nature to the way the flavonoids work. The concentrations used for stimulation were considerably varied whereas the inhibitory activity appeared to be apparent when lower concentrations of naringin were used. In our study, we used lower concentrations of naringin between 1 and 100 μM which may have been responsible for the inhibitory activity observed. The inhibitory activity of the NHDC and the NHDC ω-3 rich ester mixture appeared to be concentration dependent. Further work is needed to uncover the different mechanisms responsible for the biphasic nature of naringin activity and that of NHDC and its ω-3 rich esters.

This study has generated a class of novel bioactives – flavonoid fatty acid conjugates, demonstrating dual functionalities as lipophilic antioxidants or FAs with built-in protection against peroxidation. The methodology is feasible for large-scale manufacture with crude starting materials, and applicable to the post-harvest modification process for creating new lines of healthy ingredients with different physical properties from the parent compounds while maintaining their original functionalities.

## Author Contributions

CS contributed to the design of this study, carried out all the esterification work, drafted and finally approved the manuscript. KJ contributed to the design, execution and data interpretation of the VEGF experiment, and drafted and approved the manuscript. HW carried out all the NMR analysis and interpretation of the data. LF contributed to the grapefruit extraction, and the design and execution of the antioxidant activity experiment as well as data analysis.

## Conflict of Interest Statement

The authors declare that the research was conducted in the absence of any commercial or financial relationships that could be construed as a potential conflict of interest.

## Footnotes

1 www.novozymes.com
