# Supplementary material for: Biotransformation of Flavonoid Conjugates with Fatty Acids and Evaluations of Their Functionalities
Source: Front Pharmacol. 2017 Nov 2;8:759. doi: 10.3389/fphar.2017.00759 (PMC5673637; doi:10.3389/fphar.2017.00759)
Supplement: Supplementary file 1 [file Table_1.pdf]

Supplementary Material

Biotransformation of flavonoid conjugates with fatty acids and evaluations of their functionalities

Cynthia Q Sun*, Keryn D Johnson, Herbert Wong and L Yeap Foo

*** Correspondence:**

Corresponding Author: cynthia.sun@Callaghaninnovation.govt.nz

1 **Supplementary Table S1 – A summary of the flavonoid-fatty acids conjugates synthesized with the types of analysis and tests performed and studied, respectively.**

**Flavonoid-fatty acids esters made**	**LC-MS analysed**	**Purified**	**NMR analysed**	**DPPH assay**	**PV test**	**PA test**	**VEGF test**
Naringin lauryl ester (C12)	x	x	x	x	x	x	x
Naringin oleic ester (C18:1)	x	x	x	x	x	x	x
Naringin linolenic ester (C18:3)	x	x	-	x	x	x	x
Naringin ω-3 esters	x	x	-	x	x	x	-
NHDC ω-3 esters	x	x	x	x	x	x	x
Grapefruit extract lauryl ester	x	x	-	x	x	x	x
Grapefruit extract oleic ester	x	x	-	-	x	x	-
Grapefruit extract ω -3 esters	x	x	-	x	x	x	-

X: performed

-: not done.
